# Supplementary material for: Rice Peroxygenase-9 Negatively Regulates Production of Reactive Oxygen Species and Increases Cellular Resistance to Abiotic Stress
Source: Int J Mol Sci. 2025 Jul 18;26(14):6918. doi: 10.3390/ijms26146918 (PMC12295067; doi:10.3390/ijms26146918)
Supplement: Supplementary file 1 [file ijms-26-06918-s001.zip › ijms-3736757-supplementary.pdf]

Supplementary Materials

Rice Peroxygenase-9 Negatively Regulates Production of Reactive Oxygen Species and Increases Cellular Resistance to Abiotic stress

Anh Duc Tran[1], Kyoungwon Cho[1], Manh An Vu[1], Jeong-Il Kim[1], Hanh Thi Thuy Nguyen[2], Oksoo Han[1],*

[1] Department of Integrative Food, Bioscience and Biotechnology, Graduate School and Kumho Life Science Laboratory, College of Agriculture and Life Sciences, Chonnam National University, Gwangju 61186, Republic of Korea.

[2] Faculty of Biotechnology, Vietnam National University of Agriculture, Hanoi 12406, Vietnam.

Correspondence to: * Prof. Dr. Oksoo Han, Department of Molecular Biotechnology, Chonnam National University, Gwangju 61186, Republic of Korea. E-mail: oshan@jnu.ac.kr

Table of Contents

1. Supplementary Figures

Figure S1. Utilization of 9/13(*S*)-HOD(T)E by OsPXG9………………………………………………………………. Page 3, 4

Figure S2. Lineweaver-Burk plot of OsPXG9 kinetics in catalyzing the reaction between 9(*S*)-HODE/9(*S*)-HOTE/13(*S*)-HODE and HOOH……………………………………………………………………………………………. Page 5, 6

Figure S3. Lineweaver-Burk plot of OsPXG9 kinetics in catalyzing the reaction between 9(*S*)-HODE/9(*S*)-HOTE and CuOOH…………………………………………………………………………………………………….. Page 7, 8

Figure S4. Thin layer chromatography (TLC) analysis of OsPXG9 enzymatic reactions. ……………………………….. Page 9, 10

Figure S5. MS/MS spectra and fragmentation analysis of 9- and 13-PXG path products of OsPXG9 reaction using HOOH or CuOOH as the oxygen donor………………………………………………………………………………. Page 11-17

Figure S6. Construction of OsPXG9 overexpression (ox) lines……………………………………………………… Page 18

Figure S7. Enhancement of salt stress tolerance by the overexpression of *OsPXG9*……………………………………… Page 19

Figure S8. Schematic diagram of vector constructions for heterologous expression and generation of OsPXG9 mutant lines…… Page 20

2. Supplementary Tables

Table S1. Primer sequences……………………………………………………………………………………... Page 21

Table S2. ^{1}H NMR spectral data of peak 8 (9(*S*)-9,12,13-THODE)…………………………………………………….. Page 22

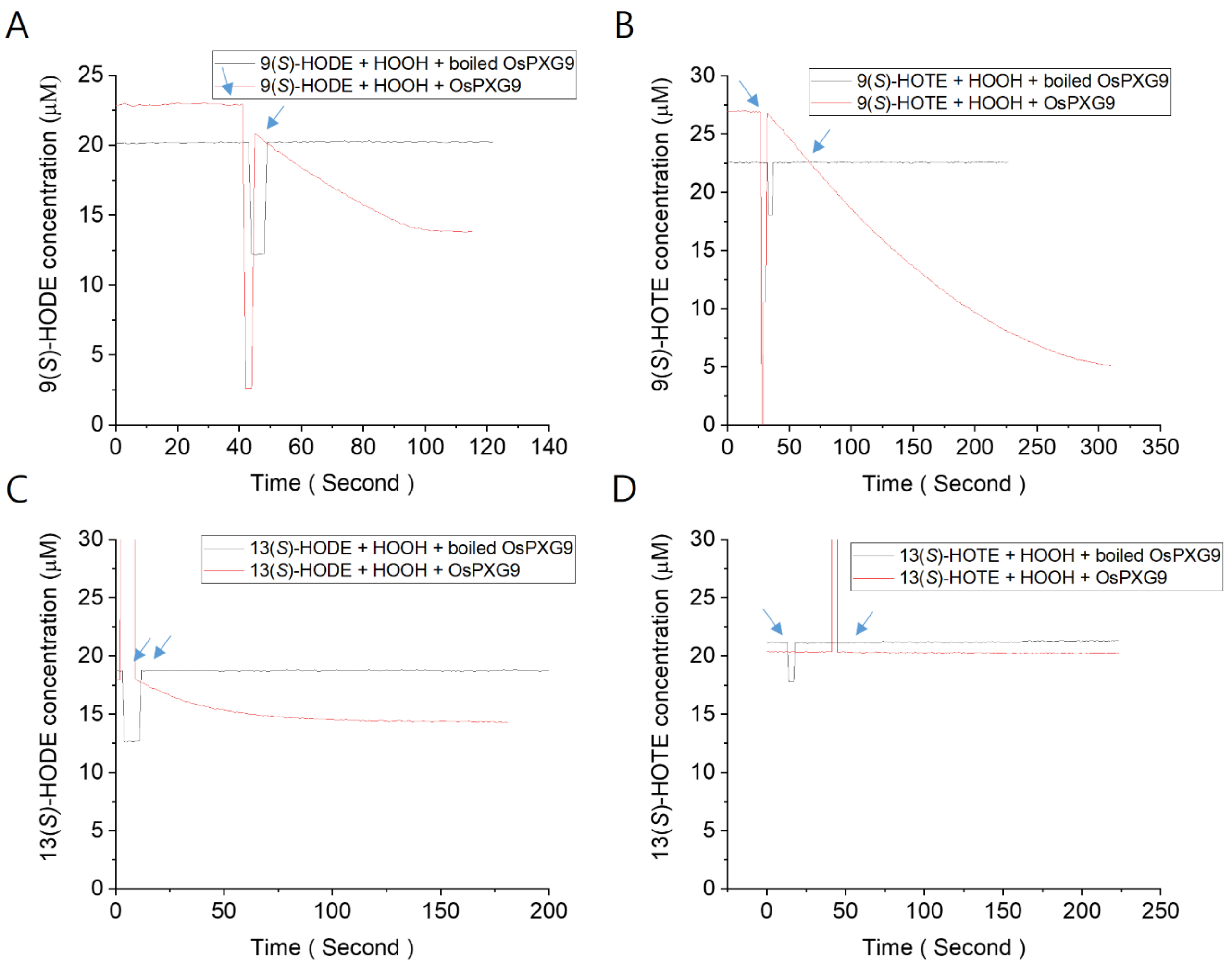

Figure S1. Utilization of 9/13(*S*)-HOD(T)E by OsPXG9. Peroxygenase activities in catalyzing the reaction of 9/13(*S*)-HOD(T)E and HOOH (A-D) or CuOOH (E-H) as substrates were checked as described in Materials and Methods. The assay monitored the disappearance of conjugated diene in 9/13(*S*)-HOD(T)E at 234 nm by adding 1 µg of OsPXG9 to the saturating concentration of substrates at the time indicated by blue arrows.

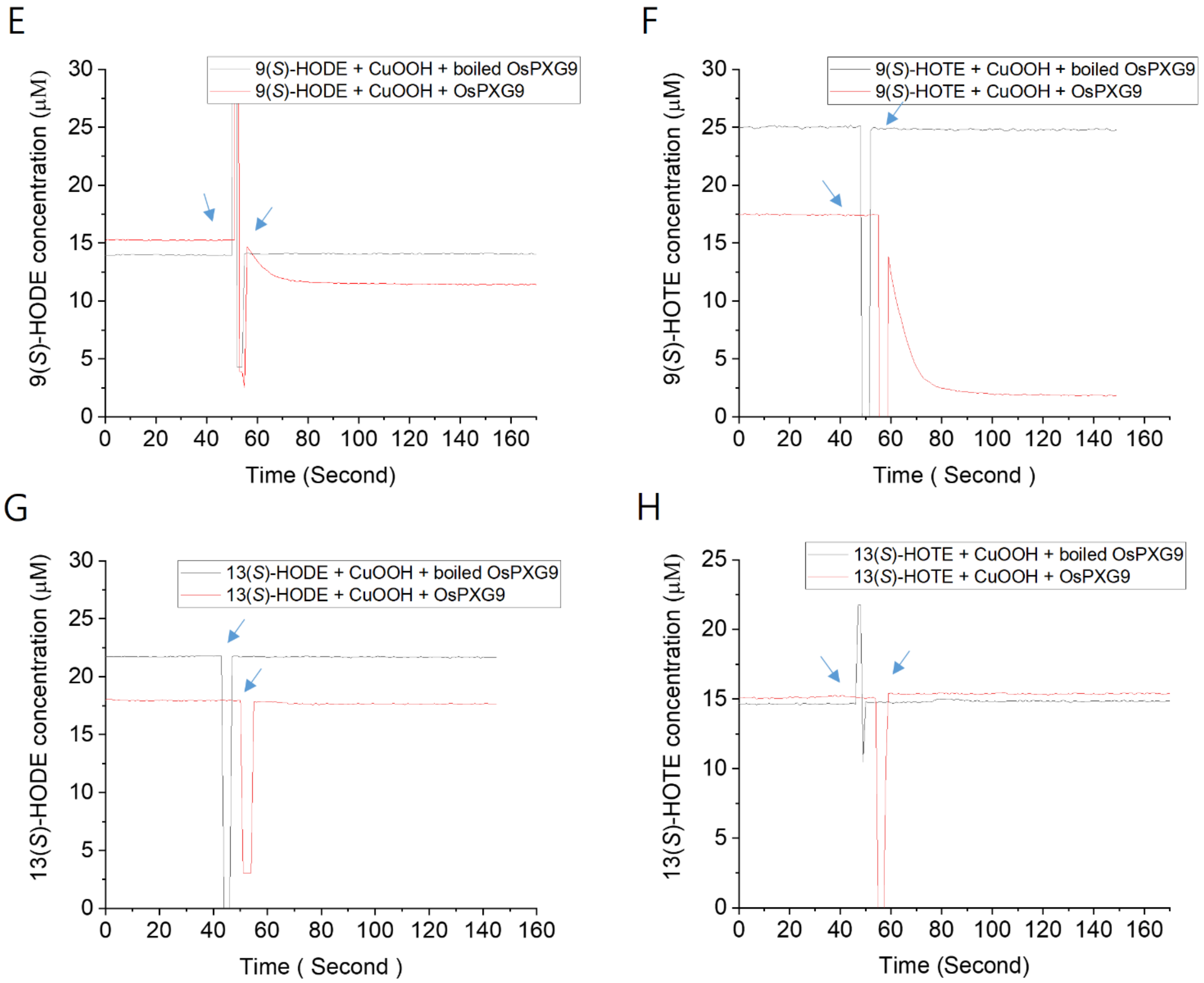
E
9(S)-HODE concentration (μM)
Time (Second)
9(S)-HODE + CuOOH + boiled OsPXG9
9(S)-HODE + CuOOH + OsPXG9
F
9(S)-HOTE concentration (μM)
Time (Second)
9(S)-HOTE + CuOOH + boiled OsPXG9
9(S)-HOTE + CuOOH + OsPXG9
G
13(S)-HODE concentration (μM)
Time (Second)
13(S)-HODE + CuOOH + boiled OsPXG9
13(S)-HODE + CuOOH + OsPXG9
H
13(S)-HOTE concentration (μM)
Time (Second)
13(S)-HOTE + CuOOH + boiled OsPXG9
13(S)-HOTE + CuOOH + OsPXG9

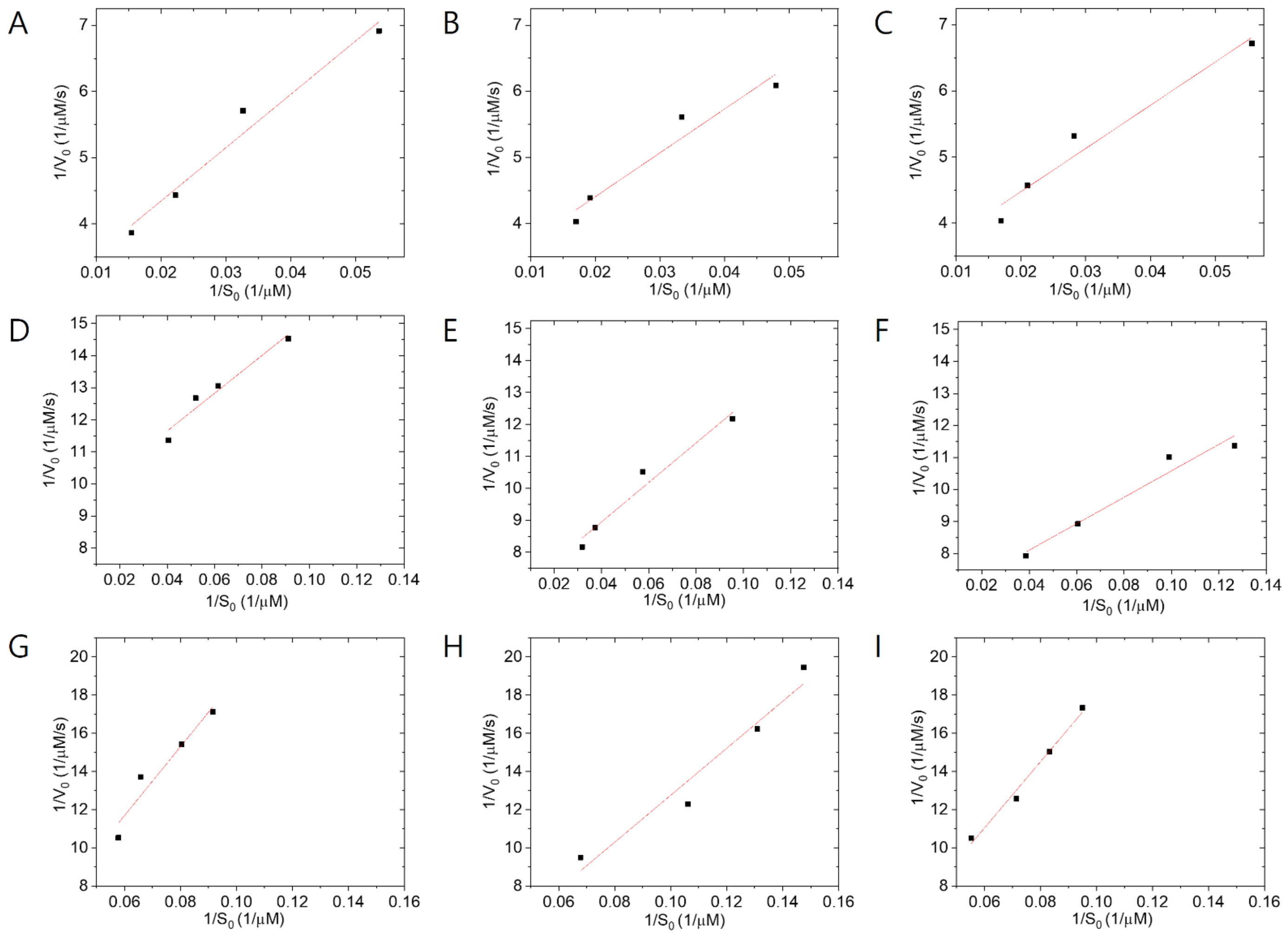

Figure S2. Lineweaver-Burk plot of OsPXG9 kinetics in catalyzing the reaction between 9(*S*)-HODE/9(*S*)-HOTE/13(*S*)-HODE and HOOH. The assay monitored the disappearance of conjugated diene of 9(*S*)-HOD(T)E or 13(*S*)-HODE by the decrease of absorption at 234 nm as described in Materials and Methods. Kinetic parameters towards the oxygen acceptors were calculated from triplicates of plots for each hydroxy fatty acid substrate: 9(*S*)-HODE (A, B, C); 9(*S*)-HOTE (D, E, F); 13(*S*)-HPOD (G, H, I) with a constant concentration of 1 mM HOOH. Kinetic parameters towards HOOH

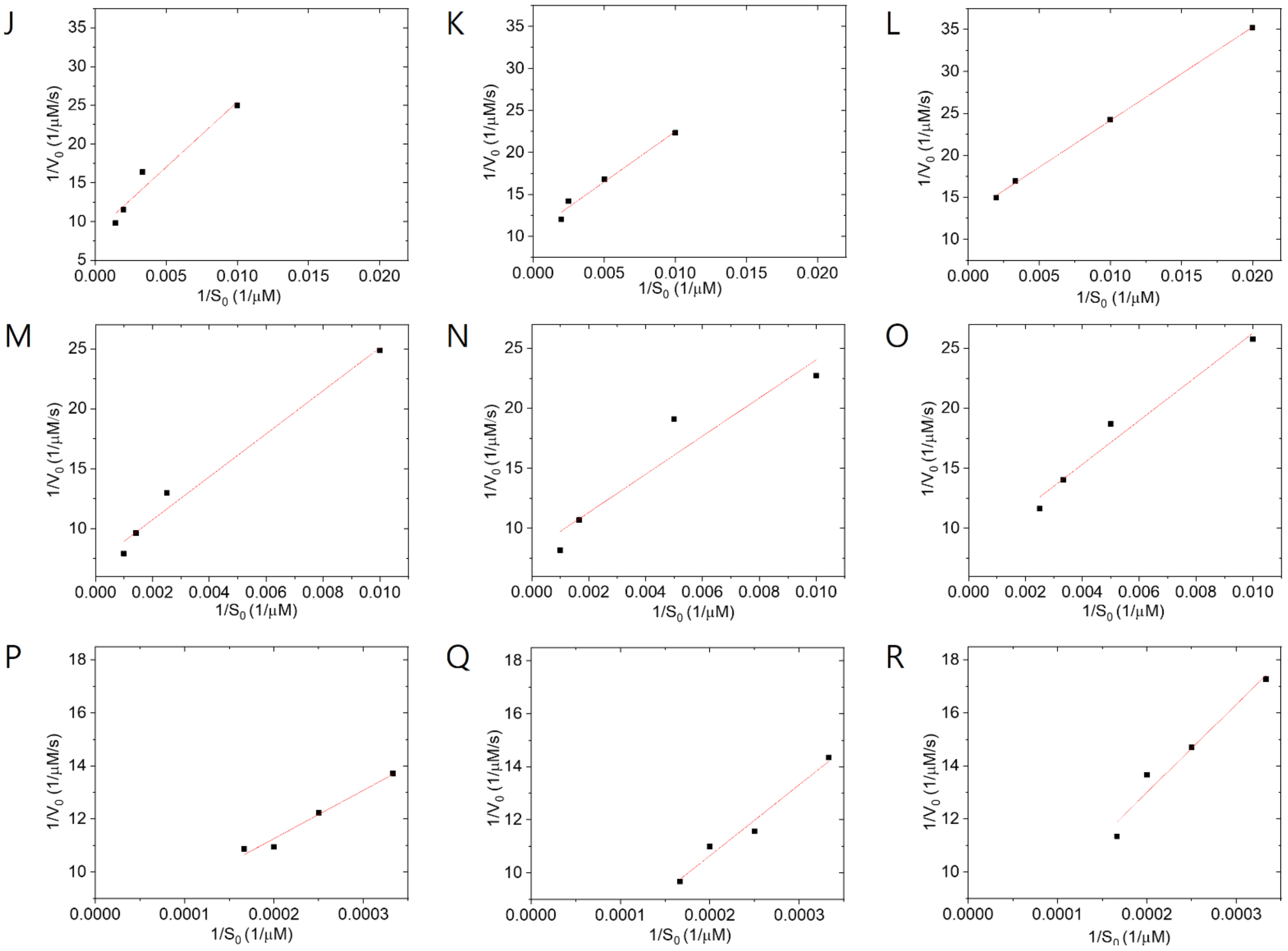

were calculated from triplicates of plots for HOOH in reaction with 9(*S*)-HODE (J, K, L); 9(*S*)-HOTE (M, N, O); 13(*S*)-HPOD) (P, Q, R) with a constant concentration of 20 μM 9(*S*)-HODE/9(*S*)-HOTE/13(*S*)-HODE.

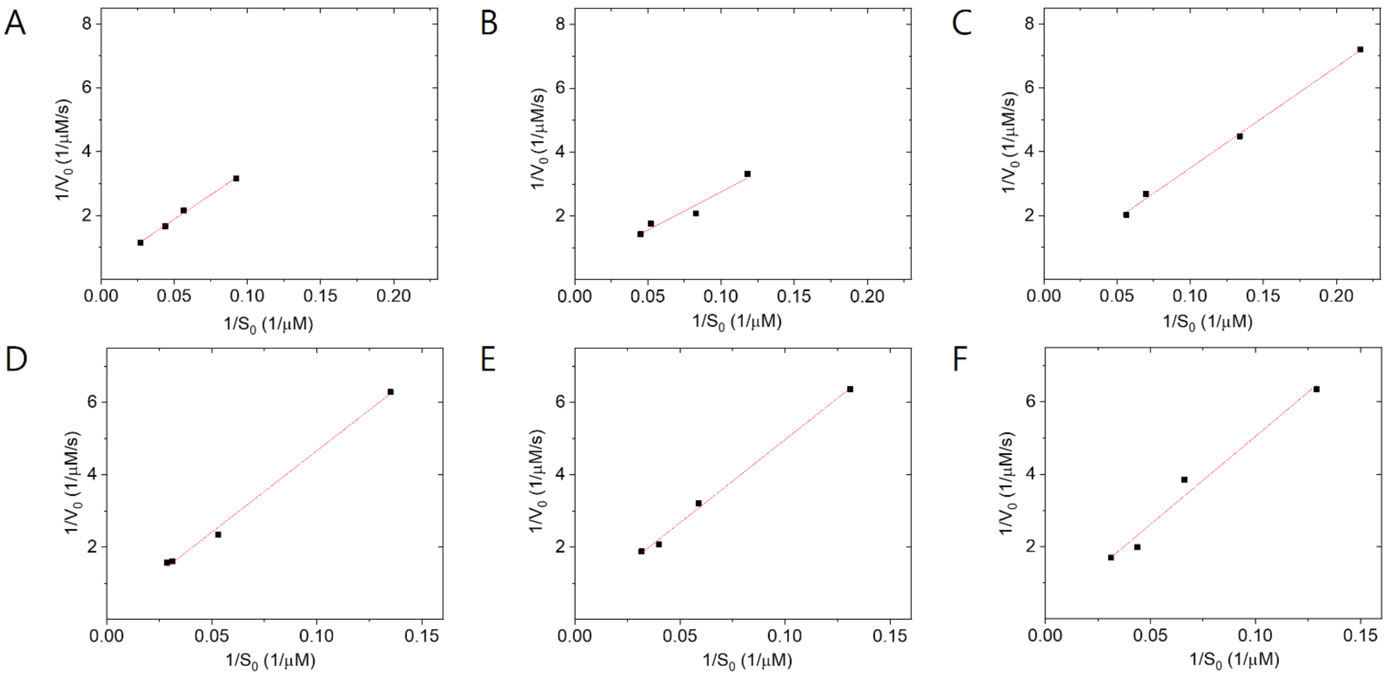

Figure S3. Lineweaver-Burk plot of OsPXG9 kinetics in catalyzing the reaction between 9(*S*)-HOD(T)E and CuOOH. The assay monitored the disappearance of conjugated diene of 9(*S*)-HOD(T)E by the decrease of absorption at 234 nm as described in Materials and Methods. Kinetic parameters towards the oxygen acceptors were calculated from triplicates of plots for each hydroxy fatty acid substrate: 9(*S*)-HODE (A, B, C); 9(*S*)-HOTE (D, E, F) with a constant concentration of 1 mM CuOOH. Kinetic parameters towards CuOOH were calculated from

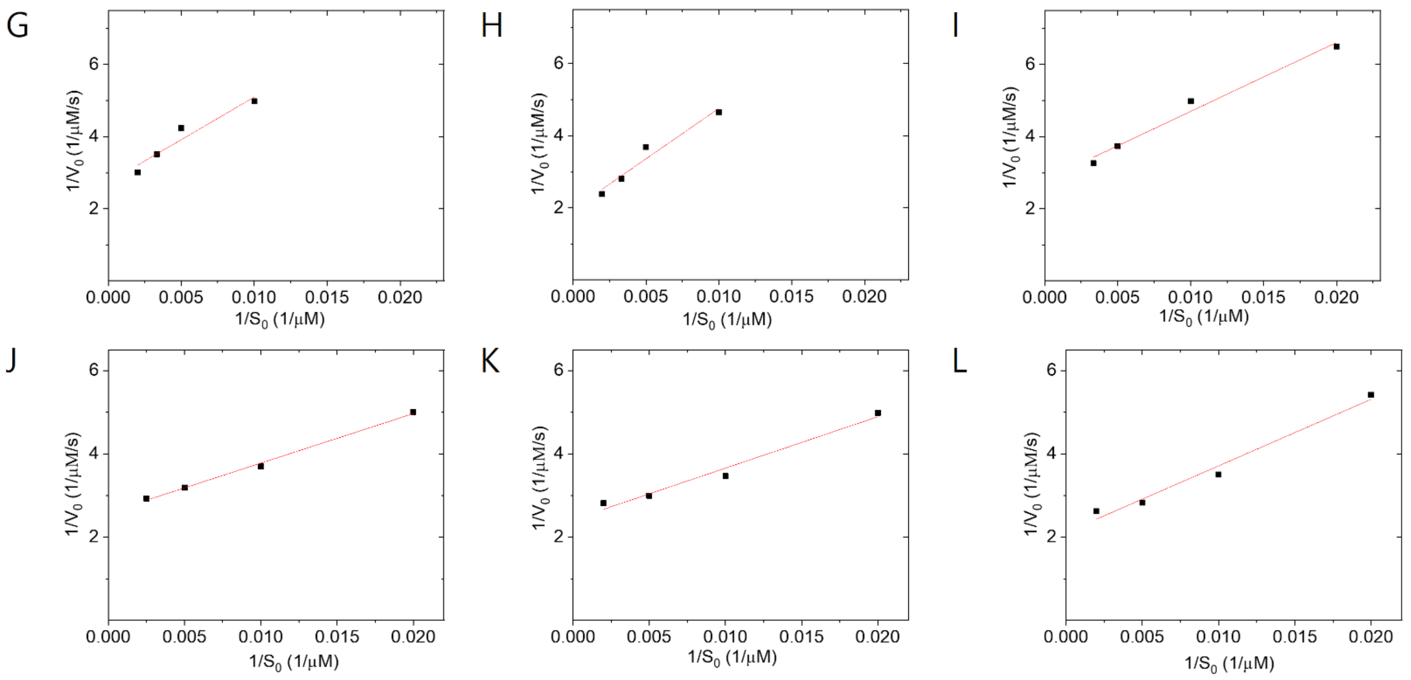

triplicates of plots for HOOH in reaction with 9(*S*)-HODE (G, H, I); 9(*S*)-HOTE (J, K, L) with a constant concentration of 20 μM 9(*S*)-HOD(T)E.

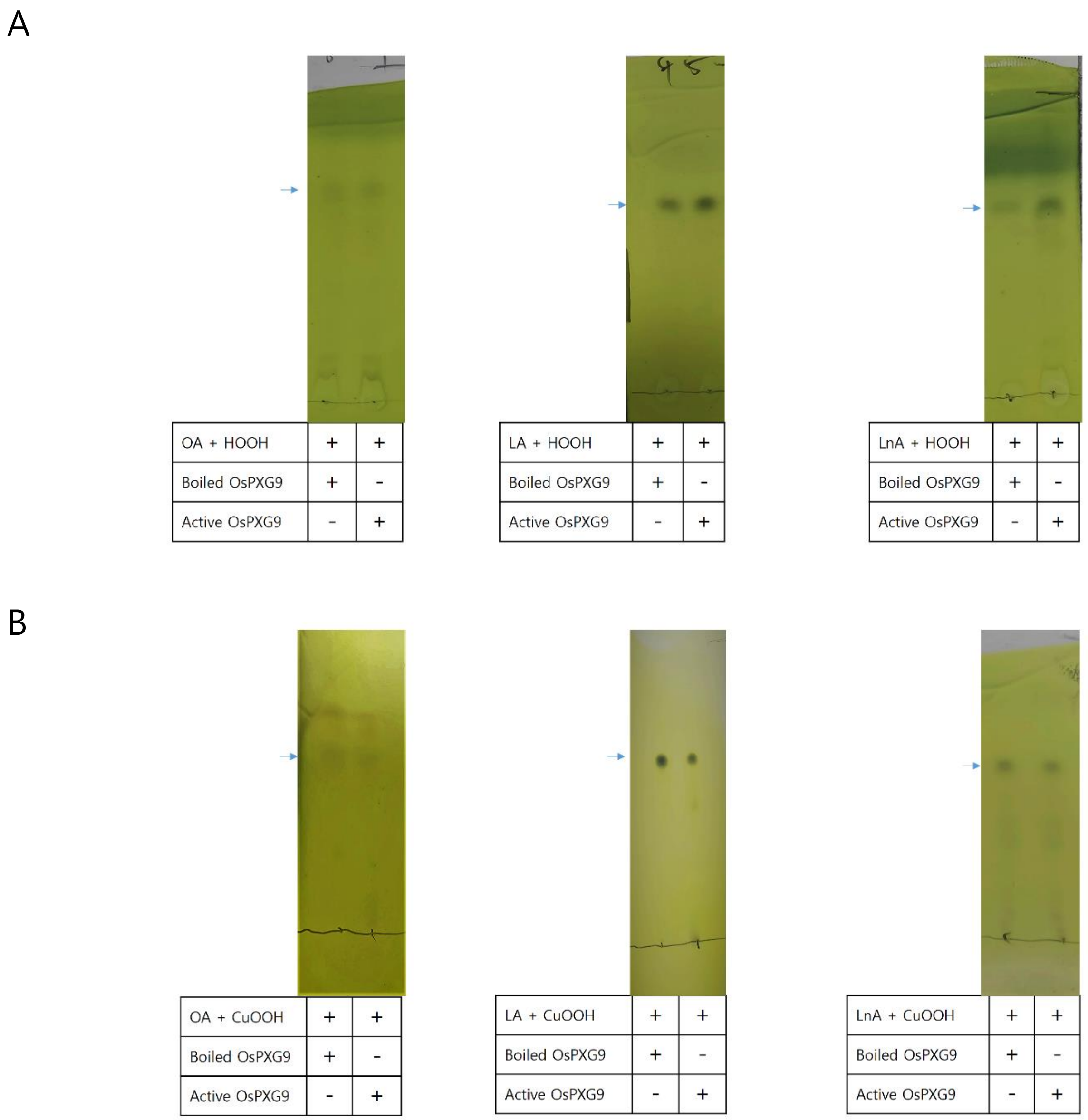

Figure S4. Thin layer chromatography (TLC) analysis of OsPXG9 enzymatic reactions. Enzyme reactions were performed with 10 μg substrates (OA, LA, and LnA for A and B; 9(*S*)-HOD(T)E for C; 13(*S*)-HOD(T)E for D) and 1 mM of HOOH or CuOOH. Boiled or active OsPXG9 (5 μg) were added and the enzyme reactions were incubated overnight. All the reaction solutions were extracted thrice by methylene chloride and the extracted solutions were concentrated by rotary evaporator. Final concentrated solutions were analyzed by TLC with the mobile phase containing Hexane:Ethyl acetate (3:2;v/v for A and B or 3:7;v/v for C and D). The substrates and catalytic products were marked by blue and black arrows, respectively.

C

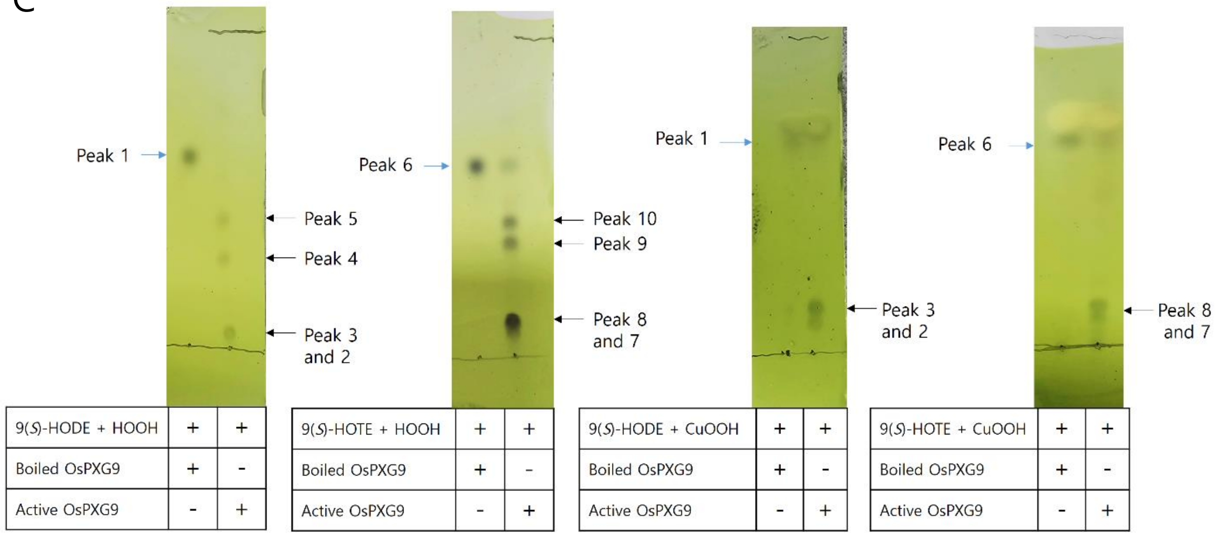

Peak 1
Peak 5
Peak 4
Peak 3 and 2
Peak 6
Peak 10
Peak 9
Peak 8 and 7
Peak 1
Peak 3 and 2
Peak 6
Peak 8 and 7
9(S)-HODE + HOOH + +
Boiled OsPXG9 + -
Active OsPXG9 - +
9(S)-HOTE + HOOH + +
Boiled OsPXG9 + -
Active OsPXG9 - +
9(S)-HODE + CuOOH + +
Boiled OsPXG9 + -
Active OsPXG9 - +
9(S)-HOTE + CuOOH + +
Boiled OsPXG9 + -
Active OsPXG9 - +

D

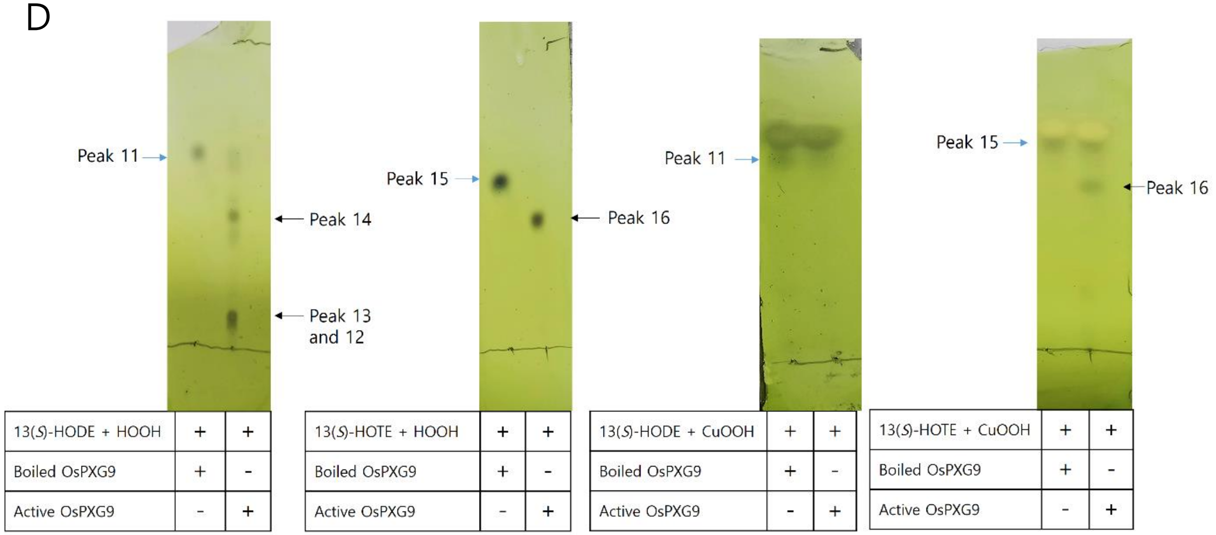

Peak 11
Peak 14
Peak 13 and 12
Peak 15
Peak 16
Peak 11
Peak 15
Peak 16
13(S)-HODE + HOOH + +
Boiled OsPXG9 + -
Active OsPXG9 - +
13(S)-HOTE + HOOH + +
Boiled OsPXG9 + -
Active OsPXG9 - +
13(S)-HODE + CuOOH + +
Boiled OsPXG9 + -
Active OsPXG9 - +
13(S)-HOTE + CuOOH + +
Boiled OsPXG9 + -
Active OsPXG9 - +

Figure S5A

Peak 1: 9(*S*)-hydroxy octadecadienoic acid

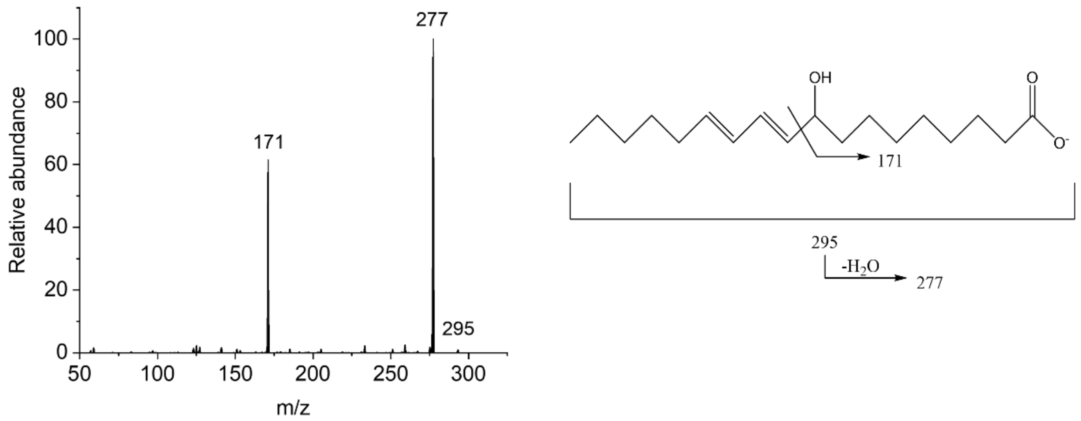

Figure S5B

Peak 2 and 3: 9(*S*)-9,12,13-hydroxy octadecenoic acid

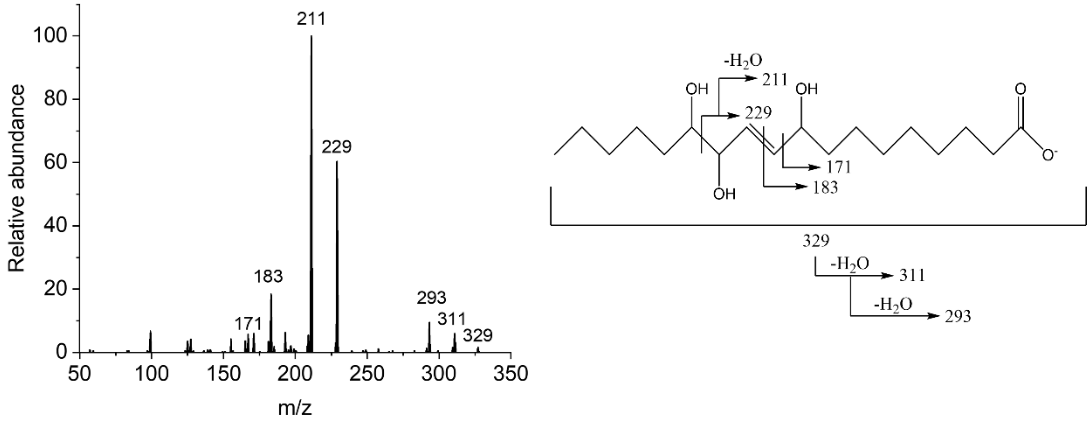

Figure S5C

Peak 4: 9(*S*)-10,11-epoxy-9-hydroxy octadecenoic acid

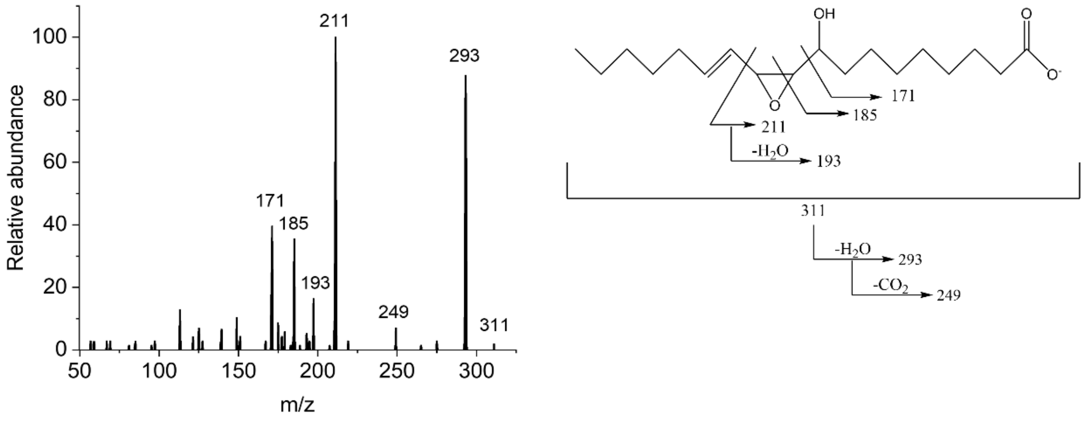

Figure S5D

Peak 5: 9(*S*)-12,13-epoxy-9-hydroxy octadecenoic acid

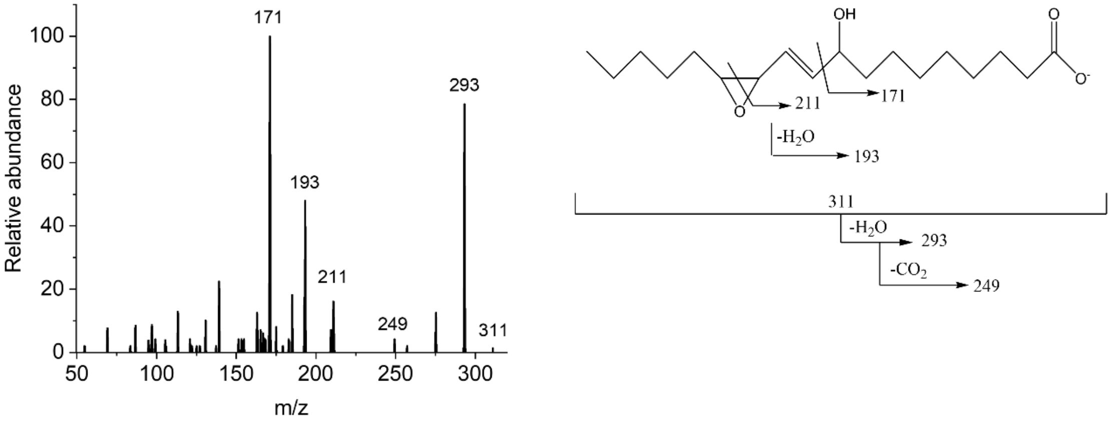

Figure S5E

Peak 6: 9(*S*)-hydroxy octadecatrienoic acid

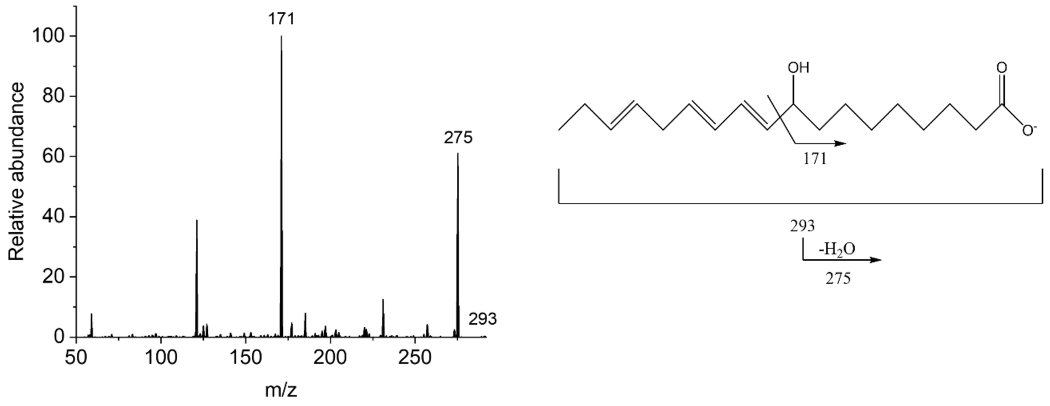

Figure S5F

F

Peak 7 and 8: 9(*S*)-9,12,13-trihydroxy octadecadienoic acid

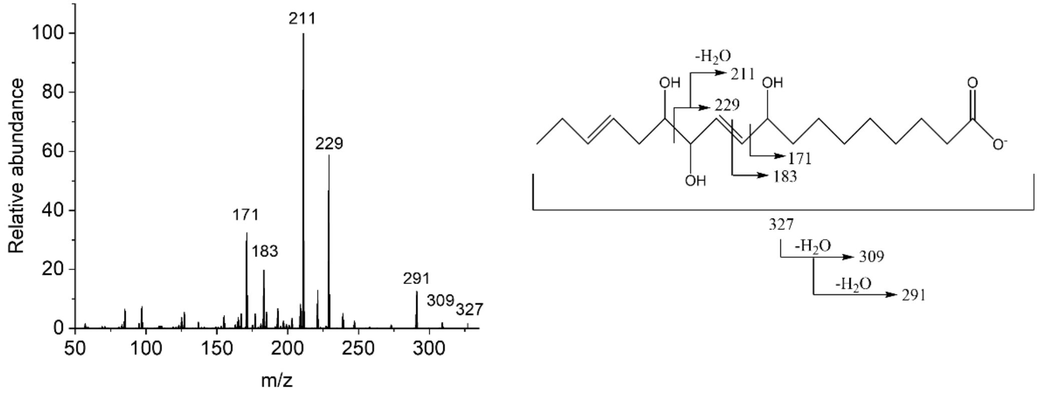

Figure S5G

Peak 9: 9(*S*)-10,11-epoxy-9-hydroxy octadecadienoic acid

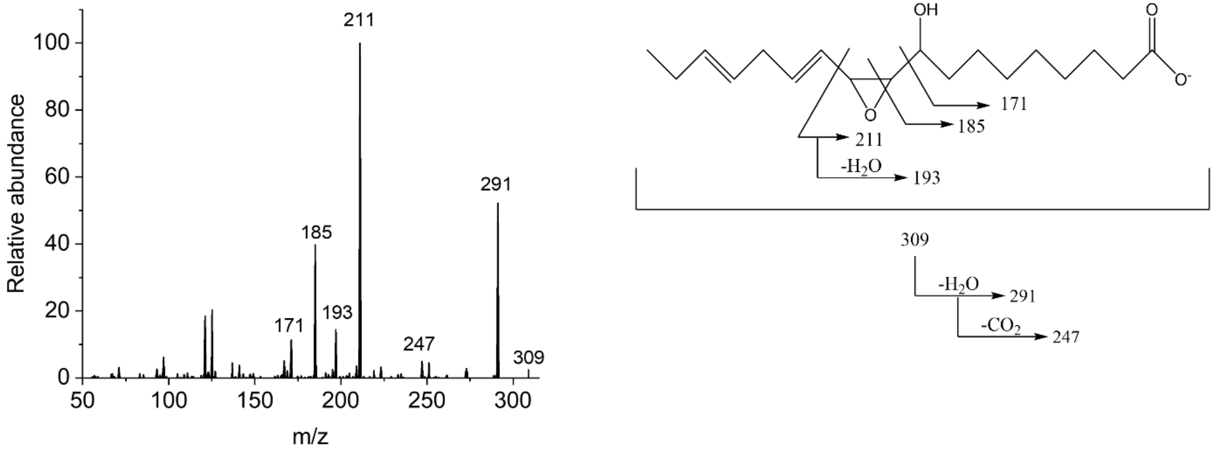

Figure S5H

Peak 10: 9(*S*)-12,13-epoxy-9-hydroxy octadecadienoic acid

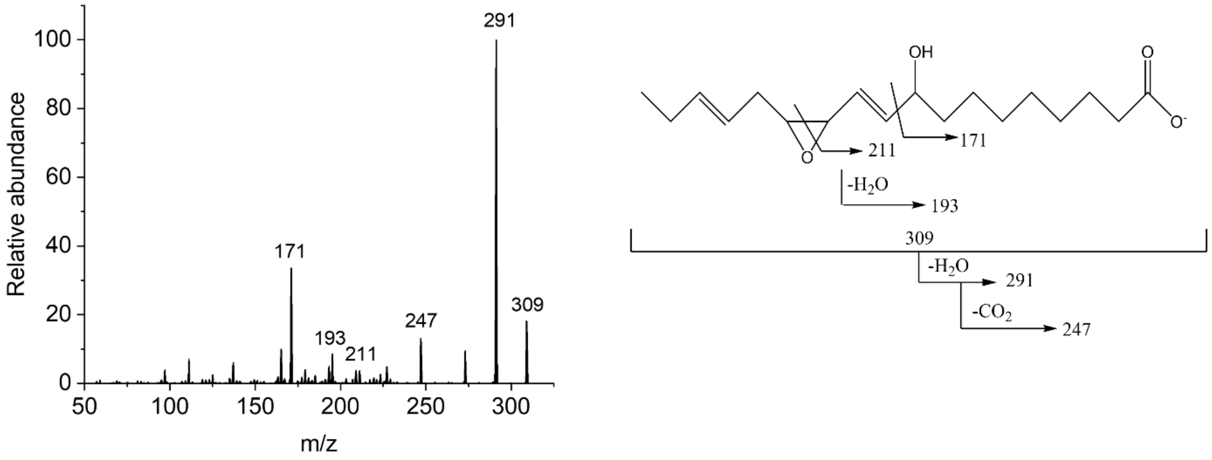

Figure S5I

Peak 11: 13(*S*)-hydroxy octadecadienoic acid

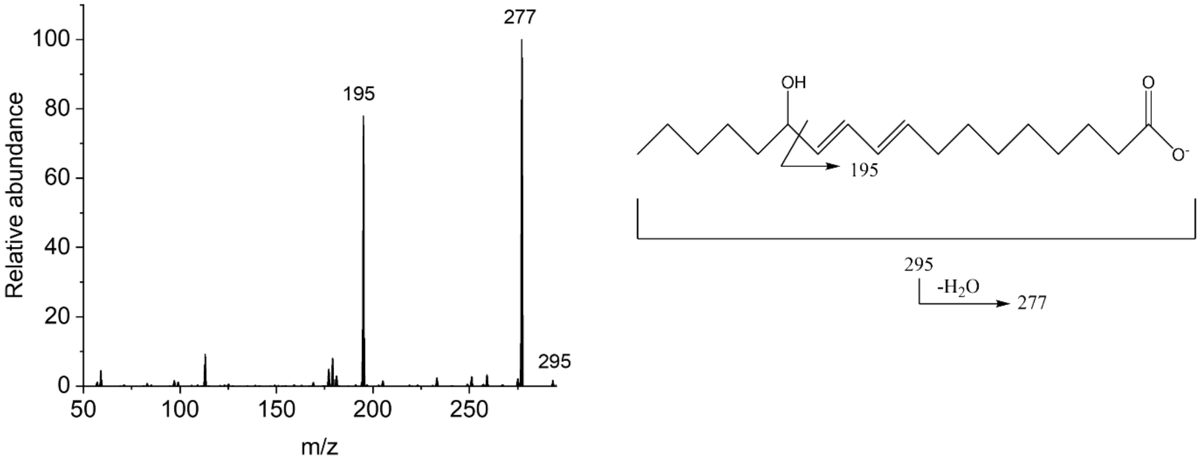

Figure S5J

Peak 12 and 13: 13(*S*)-9,10,13-trihydroxy octadecenoic acid

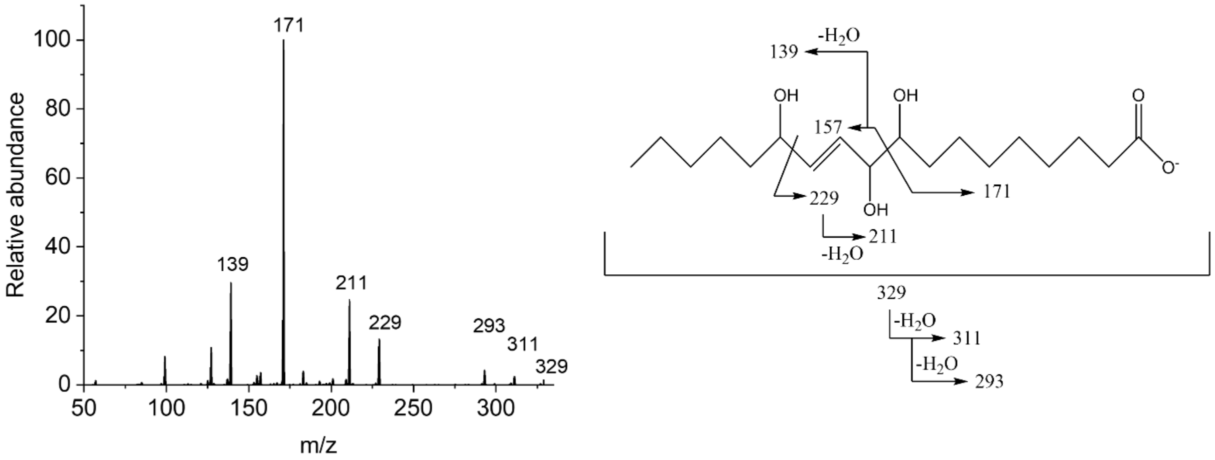

Figure S5K

Peak 14: 13(*S*)-9,10-epoxy-13-hydroxy octadecenoic acid

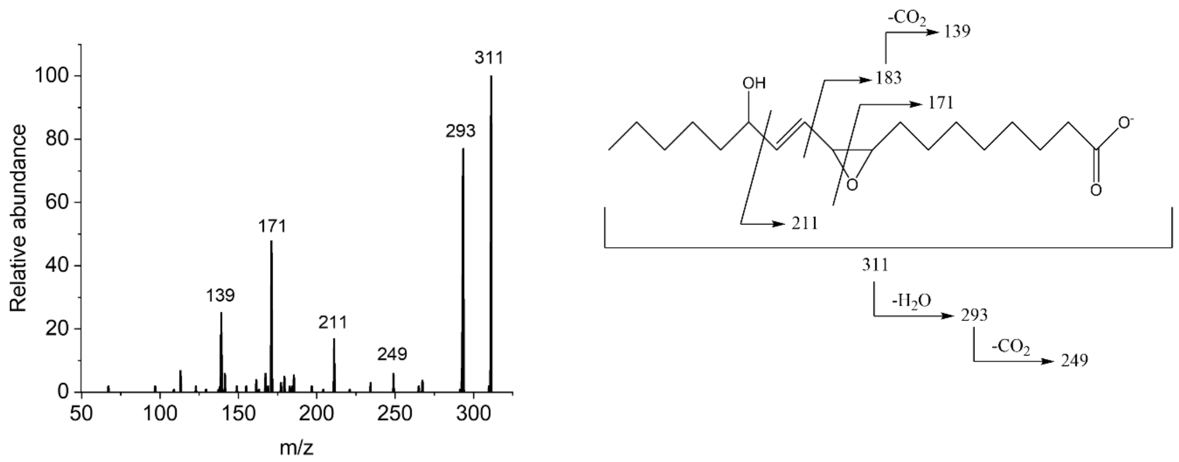

Figure S5L

Peak 15: 13(*S*)-hydroxy octadecatrienoic acid

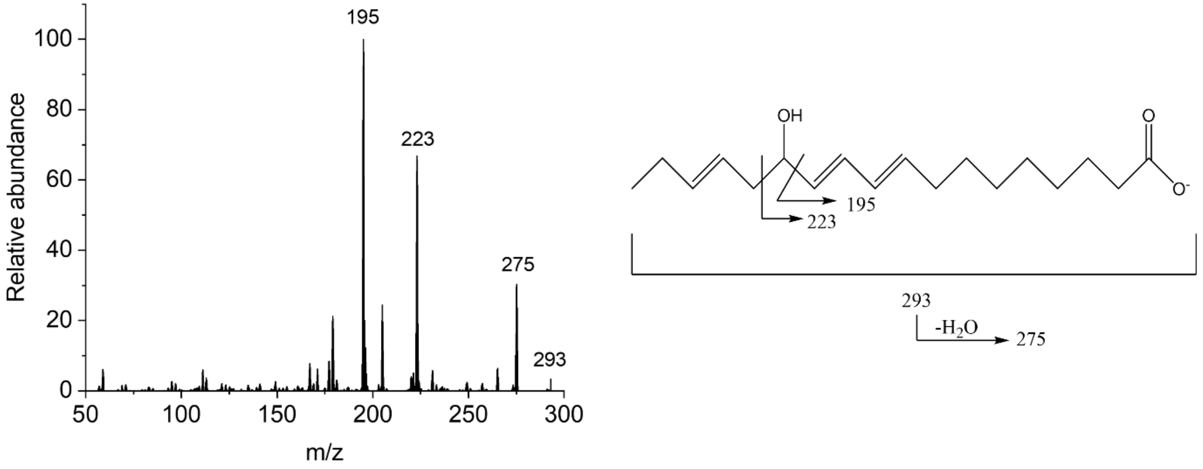

Figure S5M

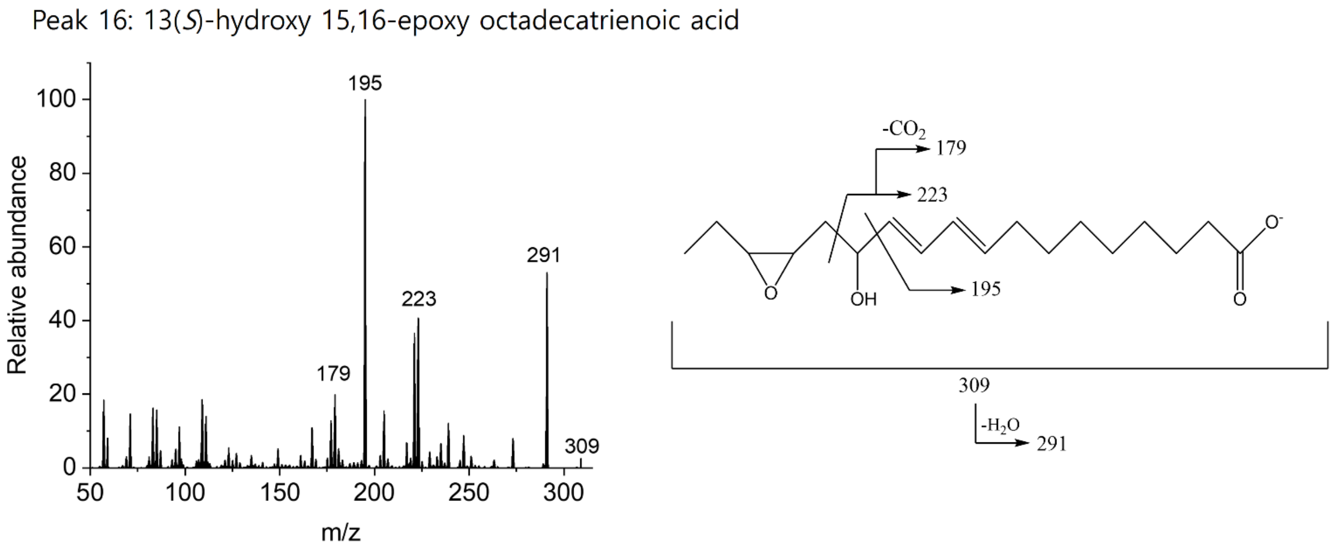

Figure S5. MS/MS spectra and fragmentation analysis of 9- and 13-PXG path products of OsPXG9 reaction using HOOH or CuOOH as the oxygen donor. (A) 9(*S*)-hydroxy octadecadienoic acid (peak 1); (B) 9(*S*)-9,12,13-hydroxy octadecenoic acid (peak 2 and 3); (C) 9(*S*)-10,11-epoxy-9-hydroxy octadecenoic acid (peak 4); (D) 9(*S*)-12,13-epoxy-9-hydroxy octadecenoic acid (peak 5); (E) 9(*S*)-hydroxy octadecatrienoic acid (peak 6); (F) 9(*S*)-9,12,13-hydroxy octadecadienoic acid (peak 7 and 8); (G) 9(*S*)-10,11-epoxy-9-hydroxy octadecadienoic acid (peak 9); (H) 9(*S*)-12,13-epoxy-9-hydroxy octadecadienoic acid (peak 10); (I) 13(*S*)-hydroxy octadecadienoic acid (peak 11); (J) 13(*S*)-9,10,13-trihydroxy octadecenoic acid (peak 12 and 13); (K) 13(*S*)-9,10-epoxy-13-hydroxy octadecenoic acid (peak 14); (L) 13(*S*)-hydroxy octadecatrienoic acid (peak 15); (M) 13(*S*)-hydroxy 15,16-epoxy octadecatrienoic acid (peak 16). Structures were identified by the interpretation of their MS/MS spectra based on mentioned metabolome databases.

A

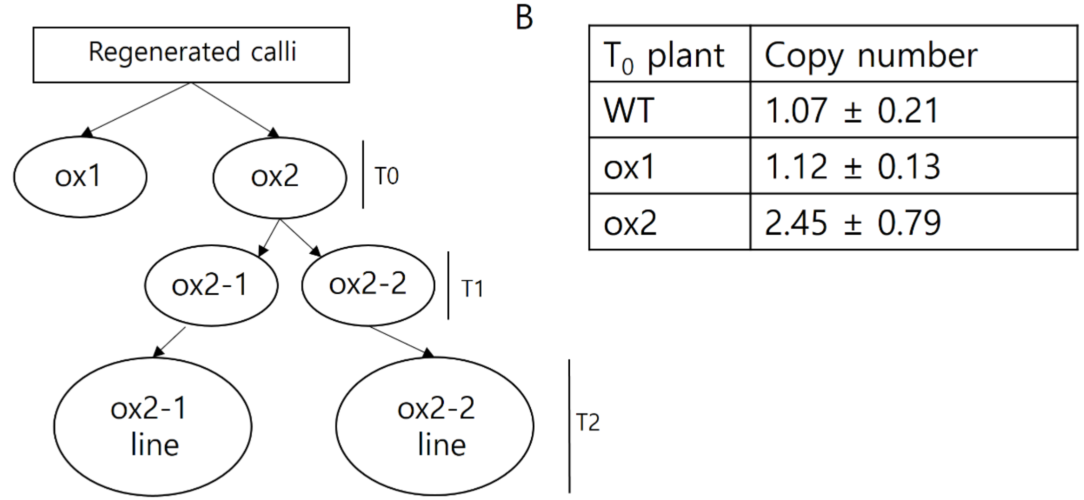

B

T_0 plant	Copy number
WT	1.07 ± 0.21
ox1	1.12 ± 0.13
ox2	2.45 ± 0.79

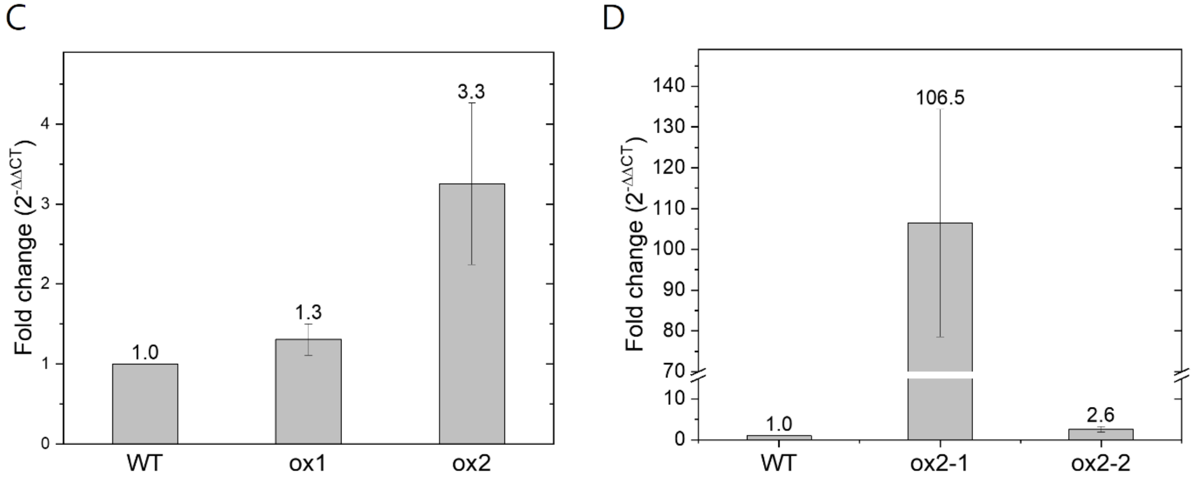

Figure S6. Construction of OsPXG9 overexpression (ox) lines. (A) Generation of OsPXG9 ox lines. (B) Copy number of *OsPXG9* in WT and two T_0 transgenic plants. (C) Relative expression levels ($2^{-\Delta\Delta CT}$) of *OsPXG9* in T_0 generation (two T_0 ox plants generated from two *Agrobacterium tumefaciens* transfected calli). (D) Relative expression levels ($2^{-\Delta\Delta CT}$) of *OsPXG9* in T_1 generation (two T_1 ox plants grown from two seeds of the ox2 plant from T_0 generation). Quantitative real-time polymerase chain reaction (qRT-PCR) assessed transcript levels of *OsPXG9* in the mature leaves of the putative transgenic plants. The experiment was triplicated with *Ubiquitin* (Os02g0161900) as the reference gene and wild-type (WT) as the reference sample. Expression of OsPXG9 in WT was used for normalization and presented as 1.0.

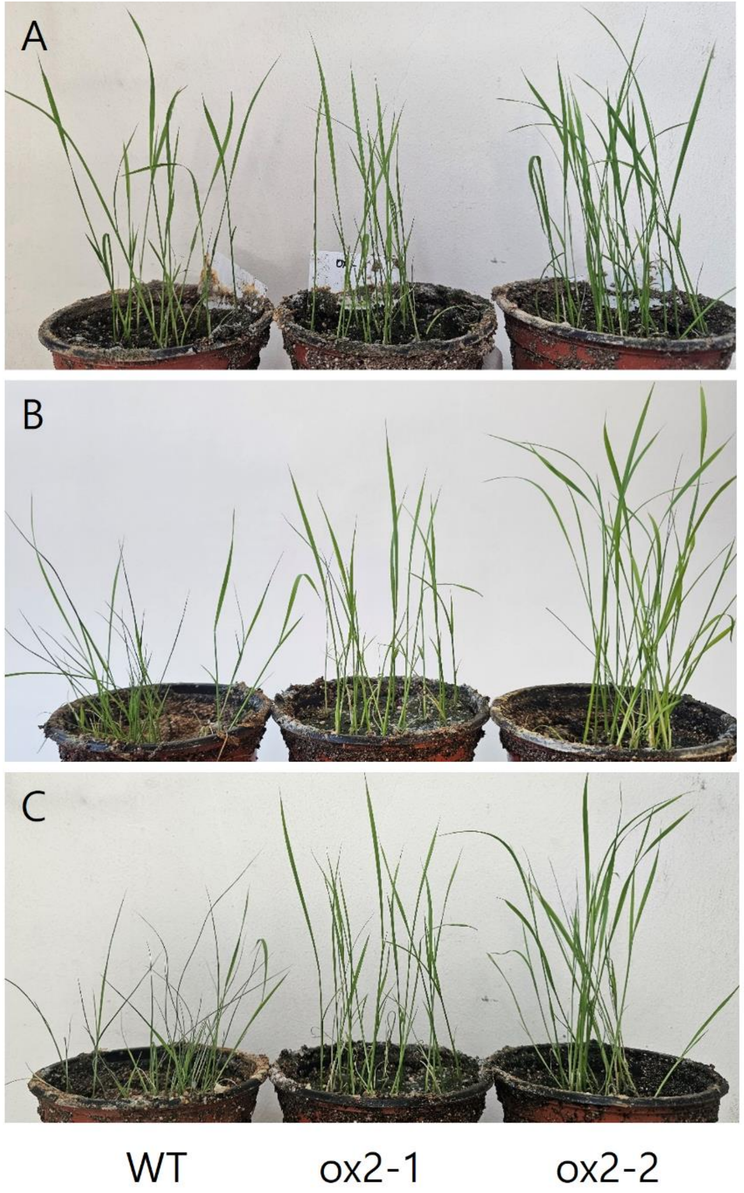

Figure S7. Enhancement of salt stress tolerance by the overexpression of *OsPXG9*. For salt stress treatment, 3-week-old plants from WT, ox2-1, and ox2-2 were subjected to 200 mM NaCl as described in Materials and Methods for 48 hours. Treated plants were allowed to recover for 96 hours in normal water. (A) Non-treated plants. (B) Treated plants after 48 hours of salt stress. (C) Treated plants after 96 hours of recovery.

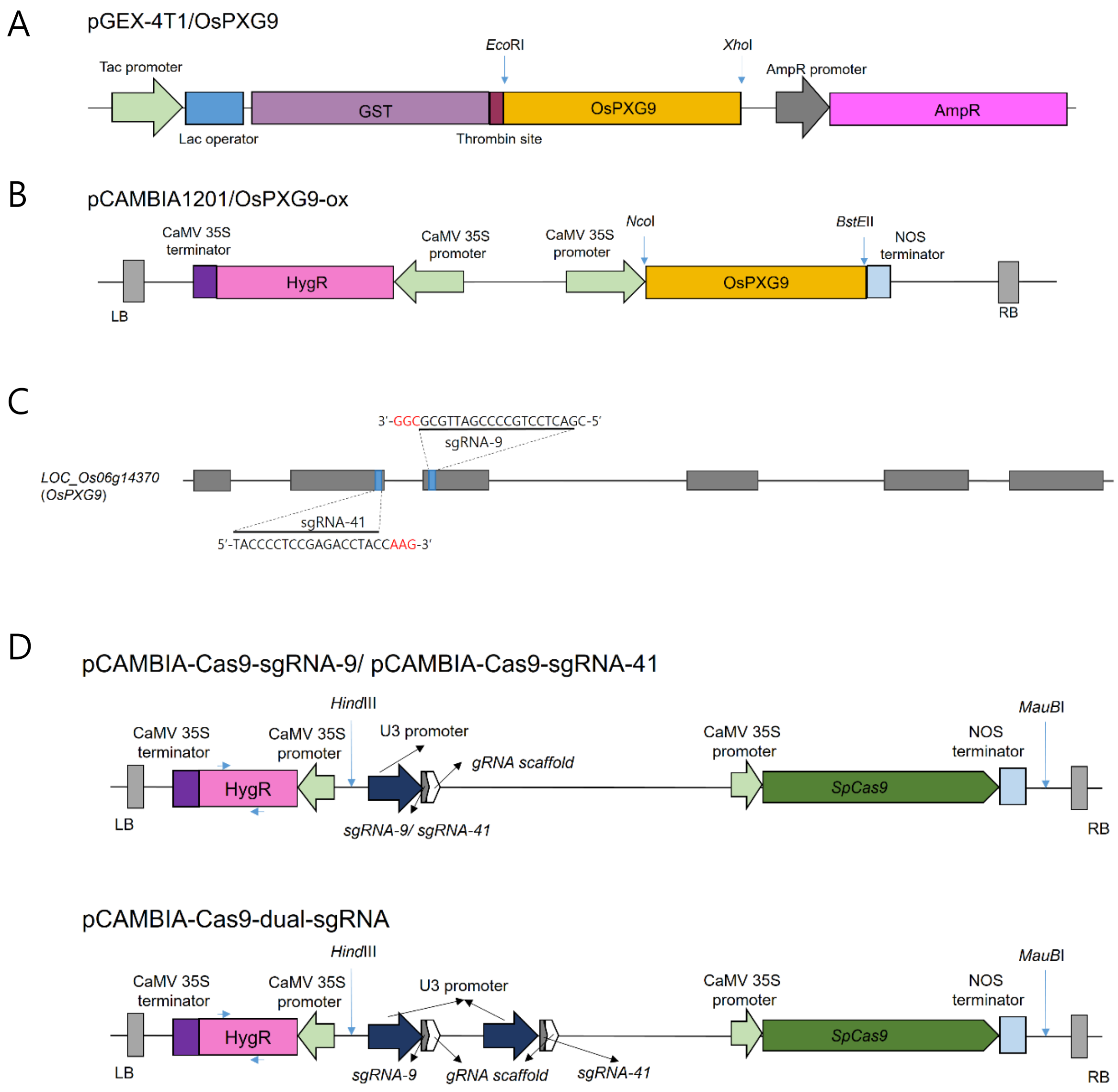

Figure S8. Schematic diagram of vector constructions for heterologous expression and generation of OsPXG9 mutant lines. (A) Construction of pGEX-4T1/OsPXG9 for heterologous expression of OsPXG9 fused with GST: cDNA of *OsPXG9* gene was amplified by PCR and inserted to commercial pGEX-4T1 vector. (B) Construction of pCAMBIA1201-OsPXG9 for generation of OsPXG9 ox lines: *OsPXG9* cDNA were obtained by PCR and cloned into the commercial pCAMBIA1201 vector. (C) Location of sgRNA-9 and sgRNA-41 on *OsPXG9* gDNA sequence. Grey boxes represent exons and black lines represent introns. (D) Construction of CRISPR-Cas9 binary vectors. The designed sgRNA-9 and sgRNA-41 were synthesized and constructed to commercial pRGE31 vector to form sgRNA cassettes (including U3 promoter, sgRNA-9/sgRNA-41/dual-sgRNA, gRNA scaffold, and SpCas9). The sgRNA cassettes were then digested directly and ligated to the pCAMBIA plasmid.

Table S1. Primer sequences

Target gene	Primer	Sequence (5'-3')	Restriction enzyme site
Vector construction of pGEX-4T1/OsPXG9			
OsPXG9 (*LOC_Os06g14370*)	F	CCG**GAATTC**ATGGCCTCCAAACCCGC	*EcoR*I
	R	CCG**CTCGAG**TTTCTTCTTAGCAGACT	*Xho*I
Vector construction of pCAMBIA1201/OsPXG9-ox			
OsPXG9 (*LOC_Os06g14370*)	F	CATG**CCATGG**ATGGCCTCCAAACCCGCG	*Nco*I
	R	GGG**TCA**C**CTC**AGTGGTGGTGGTGGTGG	*BstE*II
Vector construction of pRGE31/OsPXG9-dual-sgRNA			
	F	GGC**CCTGCAGG**AAGGAATCTTTAAACATACG	*Sbf*I
	R	CCG**GTCGAC**GCATGCACGCGCTAAAAA	*Acc*I
qRT-PCR			
OsPXG9 (*LOC_Os06g14370*)	F	CCATTCGCATCATACACGCC	
	R	CTACCCCTCCGAGACCTACC	
L-ascorbate peroxidase 1 (*OsAPX1*) (*LOC_Os03g17690*)	F	GATACCCACCATCTCCTACGCC	
	R	GGTTTCTTGTCCAAGGTCCCTC	
Catalase-A (*OsCAT-A*) (*LOC_Os02g02400*)	F	ACGAAGACGACGACGACGAA	
	R	ATGACGGTGGAGAAGCGGAC	
Superoxide dismutase [Cu-Zn] 1 (*OsSOD1*) (*LOC_Os03g22810*)	F	TTGGAAAGGGTGGGCACGAG	
	R	ACGATGCGGGCGACAGAATG	
Glutathione reductase (*OsGR*) (*LOC_Os02g56850*)	F	TGATTGAAGGGGCAGGCAGTT	
	R	CCGCCACCAAGGATTACAGC	
Determination of copy number			
OsPXG9 (*LOC_Os06g14370*)	F	CTACCCCTCCGAGACCTACC	
	R	GTCCGCCATTGATGAACACG	
Sucrose phosphate synthase (*OsSPS*) (*LOC_Os01g69030*)	F	TCTCGTGCTCTCTGCATGAC	
	R	GCCTGATCTAGCCCTACACG	

* Bold letters indicate cutting sites of restriction enzymes

Table S2. ^{1}H NMR spectral data of peak 8 (9(*S*)-9,12,13-THODE)

Protons	Chemical shift and coupling constant*
H_A	δ 4.08 (dt, J=7.2, 7.2, 1H)
H_B	δ 3.89 (dd, J=7.2, 1.6, 1H)
H_C	δ 4.26 (dt, J=7.2, 5.6, 1H)
H_D	δ 2.02 (dd, J=9.4, 3.0, 2H)
H_E, H_F, H_G, H_H	δ 5.25 (m, 4H)
$-CH_3$ and 8 $-CH_2-$	δ 1.15~1.25 (m, 19H)

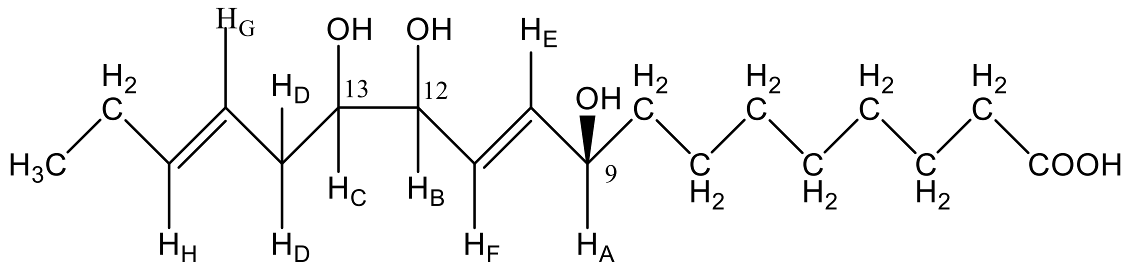

*Chemical shift in ppm, coupling constant in Hz
